# Canine Cranial Cruciate Ligament Disease (CCLD): A Concise Review of the Recent Literature

**DOI:** 10.3390/ani15071030

**Published:** 2025-04-03

**Authors:** Michael Rafla, Peilong Yang, Ayman Mostafa

**Affiliations:** 1 Department of Veterinary Clinical Sciences, College of Veterinary Medicine, Western University of Health Sciences, Pomona, CA 91766, USA; peilong.yang@westernu.edu (P.Y.); mostafaa@westernu.edu (A.M.); 2 Department of Small Animal Surgery and Radiology, Faculty of Veterinary Medicine, Cairo University, Giza 3725005, Egypt

**Keywords:** small animal surgery, cranial cruciate ligament, ligament disease, TPLO, TTA, extra-articular stabilization, intra-articular stabilization, dog

## Abstract

Objective: To review recent advancements in cranial cruciate ligament disease (CCLD) in dogs, focusing on diagnostic innovations and treatment options. Study Design: Literature review. Animals or Sample Population: Not applicable. Methods: Analysis of current literature on diagnostic tools and treatment techniques for CCLD, with emphasis on recent trends and advancements. Results: Enhanced diagnostic tools, such as Computed Tomography (CT), offer detailed anatomical insights, improving the accuracy of CCLD assessments. Treatment approaches include extra-articular techniques like lateral fabellar suture stabilization, intra-articular methods with natural and synthetic implants, and advanced surgical procedures such as Tibial Plateau Leveling Osteotomy (TPLO), Tibial Tuberosity Advancement (TTA), and arthroscopy. Recent trends favor combining techniques, such as TPLO with lateral fabellar sutures or arthroscopy with TPLO, to optimize outcomes. Intra-articular techniques, while promising, may face challenges related to graft choice and tunnel placement. Conclusions: A multifaceted approach integrating advanced diagnostics and tailored treatments enhances patient outcomes for CCLD. Ongoing advancements in both diagnostic and therapeutic strategies are crucial for effective management. Clinical Significance/Impact: This review highlights recent advancements and research in diagnostic and therapeutic strategies for CCLD, providing valuable insights for veterinary surgeons and researchers. The integration of these advanced approaches is essential for improving clinical outcomes in canine CCLD management.

## Simple Summary

Canine cranial cruciate ligament disease (CCLD) is a common orthopedic condition that leads to joint instability, pain, and reduced mobility. This review explores recent advancements in diagnosing and treating CCLD, highlighting improved imaging techniques such as computed tomography (CT) and magnetic resonance imaging (MRI) to enhance diagnostic accuracy. Treatment options range from traditional methods like lateral fabellar suture stabilization to advanced surgical techniques such as tibial plateau leveling osteotomy (TPLO) and tibial tuberosity advancement (TTA). New trends favor a combination of surgical approaches, such as TPLO with arthroscopy, to optimize patient outcomes. Post-operative rehabilitation plays a crucial role in recovery, with therapies like hydrotherapy and cold compression reducing pain and improving function. Understanding the causes of CCLD, including genetic predisposition, obesity, and biomechanical factors, is essential for prevention and effective management. This review provides veterinarians with an up-to-date perspective on the latest techniques for diagnosing and treating CCLD, ultimately improving the quality of life for affected patients.

## 1. Introduction

The purpose of this current review is to provide clinical practitioners and surgeons with an updated synthesis of advancements in diagnostic techniques, treatment options, and research findings that have emerged over the past decade. CCLD represents a significant orthopedic concern in canine medicine, affecting a wide range of breeds but disproportionately impacting larger breeds and some smaller breeds predisposed by specific conformational traits [1,2,3,4,5,6,7,8]. In addition to its clinical importance, CCLD imposes a substantial financial burden, with estimated treatment costs exceeding $1 billion annually in the United States alone [9].

The cranial cruciate ligament (CCL) is a critical structure for maintaining stifle joint stability during weight-bearing activities. It functions by limiting excessive cranial movement of the tibia relative to the femur [2,3,5,7,8]. In cases where the ligament is compromised, there is a marked increase in joint instability, leading to cartilage degradation, meniscal injury, and the eventual progression to osteoarthritis if untreated [1,2,5,7,10,11,12]. The long-term effects of untreated CCLD on joint mobility and overall stifle health underscore the importance of timely diagnosis and intervention [3,7,11,12].

CCLD is a multifaceted condition influenced by a combination of genetic predispositions, environmental factors, and biomechanical stresses [1,3,7,8,11]. A comprehensive understanding of the disease necessitates knowledge of the CCL’s anatomy and physiology, the biomechanics of the stifle joint, and the pathophysiological mechanisms underlying ligament degeneration. Additional consideration should be given to breed-specific risk factors, such as pelvic limb conformation and joint angulation, which may predispose certain breeds to ligament injuries [1,3,7,8,11].

This review seeks to integrate current knowledge by highlighting advancements in diagnostic imaging modalities, therapeutic approaches, and surgical techniques. The goal is to provide a clearer understanding of CCLD pathogenesis, improve clinical outcomes through evidence-based treatment strategies, and identify areas where further research is needed to optimize management and prevention practices for affected dogs. By addressing these key elements, this review aims to enhance clinical decision-making and improve the quality of care for canine patients with CCLD.

### 1.1. Pathophysiology and Etiology of CCLD

Cranial cruciate ligament disease is a complex condition influenced by biomechanical, genetic, and biochemical factors. The cranial cruciate ligament consists of two functional components: the craniomedial band and the caudolateral band. The craniomedial band remains taut throughout the full range of motion, making it particularly susceptible to degenerative changes and rupture. Histopathological studies have shown that the craniomedial band undergoes progressive extracellular matrix degradation, with increased expression of matrix metalloproteinases (MMPs) leading to collagen breakdown. Recent studies have demonstrated that relaxin/receptor signaling may be a primary trigger for collagen fiber degradation and collagen lysis, ultimately leading to ligament rupture [13]. Relaxin binds to LGR7 and LGR8 receptors expressed on ligament fibrocytes, initiating a biochemical cascade that upregulates matrix metalloproteinases (MMPs) and downregulates tissue inhibitors of metalloproteinases (TIMPs) [13]. This imbalance promotes extracellular matrix breakdown, leading to the progressive weakening of the craniomedial band. Immunohistochemical studies have shown a significant increase in relaxin and receptor expression in ruptured CCLs compared to normal ligaments, supporting the hypothesis that biochemical factors contribute to the degenerative process, even before mechanical failure becomes evident [13]. Additionally, the inflammatory response in the stifle joint, characterized by increased vascular permeability and synovial proliferation, may facilitate the diffusion of relaxin into the ligamentous tissue, exacerbating its degradation [13]. This suggests a multifactorial mechanism in which both intrinsic ligamentous degeneration and extrinsic inflammatory processes contribute to CCLD pathogenesis.

Historically, cranial cruciate ligament (CCL) injuries in dogs have been classified into two main categories: a degenerative condition more prevalent among middle-aged to older small and medium-sized dogs and a traumatic condition typically observed in younger, large-breed dogs [1,3,7,8,11]. Recent evidence, however, suggests that these classifications are not mutually exclusive, as the etiology of CCL injuries involves a multifactorial interplay of degeneration, biomechanics, and trauma [1,3,7]. Notably, spontaneous CCL rupture—occurring during normal physiological activities—has been documented across various breeds and ages, challenging traditional categorizations [3,7].

Trauma to the stifle joint can precipitate CCL rupture by exceeding the ligament’s tensile strength. Common mechanisms include sudden hyperextension when stepping into a pothole, sharp turns during weight-bearing activity resulting in excessive internal tibial rotation, and extreme cranial tibial thrust during landing from a jump [1,7,8]. These traumatic events highlight the biomechanical vulnerabilities of the stifle joint under certain conditions.

Approximately 80% of canine CCL ruptures are degenerative in origin, developing gradually over time. This process is characterized by progressive weakening of the ligament, often associated with aging. Studies have shown that dogs between the ages of 5 and 7 years are particularly susceptible to degenerative CCL rupture [7,8]. Degenerative joint disease (DJD) and joint instability frequently accompany ligament deterioration, which can progress from partial tears to complete rupture [3,7,11].

Conversely, acute CCL rupture is more common in dogs under 4 years old and is typically caused by traumatic injuries. These injuries often occur during activities such as running, jumping, or sudden directional changes, where excessive forces are applied to the stifle joint. The clinical signs of acute CCL rupture, such as severe lameness and joint pain, manifest immediately following the incident [7,8].

Age-related changes significantly contribute to CCL rupture. The ligament undergoes structural alterations as dogs mature, particularly in larger breeds. Histopathological studies have demonstrated fibroblast depletion, collagen disorganization, and the accumulation of areolar tissue within the ligament, all of which compromise its mechanical integrity and increase susceptibility to rupture [8]. Additionally, larger breeds weighing more than 15 kg exhibit more pronounced degenerative changes, likely due to greater biomechanical stress on the stifle joint [6,7,8,11].

The instability caused by CCL rupture initiates a cascade of degenerative changes within the stifle joint. These include meniscal injury, osteophyte formation, synovitis, and articular cartilage breakdown characterized by disorganized collagen fibrils and chondrocyte hypertrophy [7,8,11,14,15]. Such alterations underscore the importance of early surgical intervention to prevent irreversible joint damage and preserve long-term function.

Conformational abnormalities in the pelvic limbs play a significant role in the development of CCL disease. Internal torsion of the distal femur and increased angulation of the proximal tibia are key anatomical factors that enhance cranial tibial thrust during weight-bearing, contributing to chronic ligament stress and eventual rupture [1,3,7,11]. These abnormalities are often accompanied by conditions such as patellar luxation and hip dysplasia, which further destabilize the joint [1,3,7,11,16]. Identifying and addressing these conformational predispositions can facilitate early intervention, potentially mitigating the severity of CCL disease in predisposed breeds [3,5,7,8,11].

Understanding the interplay of age, biomechanics, and anatomical predispositions is crucial for developing targeted preventive and therapeutic strategies for CCL disease. By tailoring interventions to the specific needs of affected dogs, veterinarians can improve clinical outcomes and enhance the quality of life for canine patients with this complex condition (Table 1).

### 1.2. Predisposing Factors

Certain dog breeds are particularly predisposed to cranial cruciate ligament (CCL) injuries due to their size, conformation, and genetic factors [3,7,11]. Large and overweight dogs are notably susceptible to CCL rupture, as their increased body weight places greater stress on the joints, accelerating degenerative processes that weaken the ligament [3,7,8]. Breeds commonly affected include Labrador Retrievers, Newfoundlands, Rottweilers, Neapolitan Mastiffs, Saint Bernards, Chesapeake Bay Retrievers, American Staffordshire Terriers, Akitas, Boxers, and Bulldogs [7,8]. Specific conformational anomalies in breeds such as Chow Chows, Rottweilers, American Staffordshire Terriers, Boxers, and Saint Bernards, including hyperextended pelvic limbs and open joint angles, contribute to ligament weakening and predispose them to CCL rupture [8,17]. Additionally, these breeds frequently exhibit hip dysplasia, which further stresses the ligaments and influences the quadriceps mechanism [7,8].

In smaller breeds, CCL injuries are often associated with excessive stress caused by tibial instability linked to conditions such as patellar luxation, particularly in severe cases (grade IV) [8,11,16]. Dogs with genu varum, an abnormal conformation of the pelvic limbs, also experience increased strain on their ligaments. Obesity exacerbates these issues by elevating concentrations of inflammatory mediators like adipokines, which promote degenerative changes in ligament tissues [3,7].

Genetic factors also play a significant role in disease susceptibility. Emerging evidence suggests that parity in female dogs may influence their risk of CCL disease. A lifetime cohort study of Rottweilers revealed that parous bitches had a 94% lower risk of CCL rupture compared to nulliparous bitches, even after adjusting for various factors [18]. This finding underscores the need for further research to understand the underlying mechanisms and explore parity’s potential impact on other health conditions in dogs.

The breakdown of the CCL might also be influenced by immune-mediated processes [1,3,7]. Joint inflammation can make the ligament more vulnerable by triggering ongoing tissue degradation. Elevated levels of autoantibodies against collagen in dogs with cruciate disease suggest a possible autoimmune component [1,3,7,8]. These autoantibodies may initiate inflammatory responses, activating macrophages and contributing to the breakdown of the ligament’s extracellular matrix. Enzymes such as matrix metalloproteinases (MMPs), including MMP-1, MMP-8, and MMP-13, as well as cathepsin K and tartrate-resistant acid phosphatase (TRAP), are implicated in collagen degradation, further compromising ligament integrity and potentially leading to rupture [3,7,8].

Obesity remains a significant factor contributing to degenerative changes and weakening of ligaments, primarily through the release of pro-inflammatory adipokines by adipocytes [3,7,8]. Adiponectin, a protein encoded by the “ADIPOQ” gene, regulates enzymes involved in ligament tissue remodeling. Variations in the ADIPOQ gene can alter adiponectin levels, potentially affecting ligament strength and susceptibility to disease [8].

Lack of physical activity further exacerbates ligament vulnerability by causing muscle and tendon atrophy, thereby reducing joint stability during movement [8]. Weak muscles and impaired neuromuscular control increase ligament strain, which can lead to gradual wear and tear. Structured training programs designed to improve proprioception and muscle function have been shown to effectively reduce ligament injuries in dogs [7,8].

These factors highlight the complex relationship between breed predisposition, obesity, genetic influences, and immune-mediated mechanisms in CCL disease. A comprehensive understanding of these interrelated factors is essential for developing effective prevention and treatment strategies. Addressing these underlying causes can significantly improve the long-term health and mobility of affected dogs (Table 2).

## 2. Diagnosis of Canine CCLD

### 2.1. Symptoms of CCLD

Although the clinical manifestations of cranial cruciate ligament disease (CCLD) can differ, they usually consist of a combination of symptoms indicating joint instability and discomfort [7,19]. The sit position, in which the dog sits with its rear leg outstretched rather than tucked beneath its body to indicate discomfort and instability, is another typical indicator [7]. In addition to having a restricted range of motion of the injured joint, dogs with CCLD may find it difficult to utilize the leg normally [7,19]. Crepitus, a crackling sound produced when bones scrape against one another as a result of an unstable joint, is another important sign [7].

Pain plays a major role in CCLD, especially when it involves the stifle joint. To keep their suffering from becoming worse, dogs in pain try to limit their physical activity [7,20]. In addition to often displaying limited extension or mobility in the affected leg, dogs with CCLD may often walk with a noticeable limp or an abnormal gait [7,15,21]. Feeling stiff after exercise is another typical symptom [7,8]. The afflicted area may also become swollen and feel thick or firm on joint manipulation, which could indicate inflammation and fluid buildup [5,7,22]. To ease the pain in the leg, dogs with CCLD may redistribute their weight to one side when standing [5,7]. The improper alignment of the joint following a CCL rupture may result in more harm, pain, and edema, all of which may accelerate the onset of degenerative joint disease (DJD) [7,8,11,15,22]. If the meniscus is also torn, the dog may move with a cracking or snapping sound. The symptoms worsen by this additional injury, which also can cause further complications [7]. According to the extent of the CCL tear (partial versus complete) and the associated structural diseases (such as synovitis, medial meniscus injury, and osteoarthritis), the lameness may vary from mild to minimal or non-weight-bearing lameness [6,7,11].

### 2.2. Physical Exam

Palpation and manipulation tests are essential for determining the level of ligament damage and musculoskeletal anomalies in a dog suspected of having a CCL injury [3,7]. Palpation involves assessing the dog while standing to spot signs of a CCL injury, such as limb asymmetry, discomfort, edema/joint effusion, or muscle atrophy [3,7,15]. Chronic instances frequently show cranial displacement of the tibial crest, thickening of the joint capsule (particularly medially), and atrophy of the quadriceps muscles [3,7]. Additional probing in lateral recumbency is necessary to differentiate between joint effusion and fibrotic thickening, which is important for staging the injury as acute or chronic [3,7]. In order to identify cranial tibial translation relative to the corresponding femur (i.e., stifle instability) and confirm CCL rupture, a common manipulative test, named the cranial drawer test, is carried out by creating active tibial translation with the stifle being slightly flexed [3,7,14]. False-negative results, however, might occur, especially in painful, nervous, or tense large-breed dogs. Mild sedatives or tranquilizers may then be recommended [7,22]. Another test to evaluate joint stability is the tibial compression test performed by creating gastrocnemius compression on the tibial crest while flexing the hock joint gently. A positive test is indicated by passive minimal cranial translation of the tibial crest in CCL rupture. This particular test could be helpful when patient size or technical difficulties render the result of the cranial drawer test unclear [7].

### 2.3. Synovial Fluid Analysis

A synovial fluid analysis can help rule out immune-mediated causes of CCL rupture and distinguish between acute and chronic inflammatory processes [7,8]. Evaluations of fluid color, volume, transparency, viscosity, and cell counts are all included in the analysis [7,8]. Hemarthrosis is a common presenting feature of acute trauma, but degenerative joint disease may manifest in chronic situations [7]. The diagnosis can be further refined by the identification of cells and debris, and thus treatment options can be guided accordingly. Acute trauma is suggested by neutrophil predominance, although synovial hypertrophy and increased mononuclear cells may be seen in chronic instances [7,8,16]. In addition, biochemical indicators including MMPs, CRP, and adipokine levels aid in the long-term monitoring of joint health and inflammatory conditions [7,8,18,20].

### 2.4. Radiography

When it comes to diagnosing and planning therapy for CCL injuries, radiography is a vital diagnostic imaging modality because it provides important information on joint morphology, alignment, and secondary changes [7,11,16]. When assessing the stifle joint, mediolateral and craniocaudal views are typically used to look for indications of a diseased CCL, such as joint effusion, osteophyte formation, and changes in joint space [12,14,23]. Radiologists and veterinarians specifically search for signs of joint instability, such as chronic joint alterations associated with osteoarthritis, possible cranial displacement of the tibia with respect to the femur (with or without stress stifle radiography), and/or steepness of the tibial plateau [12,14,23,24]. To confirm the diagnosis and direct surgical decisions, further images, such as the stress radiograph for joint laxity assessment and the skyline view for patellar alignment, may be used. In order to establish a baseline for tracking the course of the disease and analyzing the effectiveness of post-surgery treatment, radiographic interpretation focuses on evaluating these parameters in order to determine the degree and severity of CCL injury [12,14,23,24]. The tibial plateau angle (TPA), which gauges the tibial plateau’s slope, is one important metric [14,24,25]. Increased cranial tibial thrust is frequently linked to an increased TPA, which can lead to CCL damage [14,15,24,26]. Precise measurement of TPA aids in the planning of surgical procedures, including TPLO, and assesses how well they stabilize the stifle joint and lessen the mechanical strain on the CCL [15,19,25,26]. Understanding TPA and its implications can improve therapeutic outcomes and guide prophylactic measures for dogs susceptible to CCL disease.

Recent studies have highlighted the importance of accurate measurements and the impact of dog compliance on these assessments. For instance, a study evaluating stifle goniometry in awake dogs with CCL disease compared to healthy dogs found excellent intra- and interobserver reliability for most measurements [21]. However, interobserver reliability for stifle extension in healthy dogs was only fair, possibly due to variability in dog compliance and differences in measurement conditions (e.g., awake vs. sedated) [21]. This variability underscores the need for careful consideration of dog behavior and potential use of sedation to minimize measurement variability [21]. Such considerations are crucial for ensuring accurate TPA assessments and effective surgical planning.

Conventional stifle radiographs can reveal indirect signs of CCL rupture, e.g., effusion obliterating the infrapatellar fat pad or osteophytes, but stress positioning can enhance diagnostic yield [27,28]. Tibial compression (stress) views—taken with the hock flexed to induce cranial tibial subluxation—are particularly useful in detecting instability even when a joint drawer is not obvious [27]. In dogs with CCL rupture, a “Cazieux-positive” sign (spontaneous cranial tibial subluxation on a lateral radiograph) is highly specific. A recent study found that if the tibia is visibly displaced cranially relative to the femur on a standing lateral radiograph, it virtually guarantees a complete CCL tear—positive predictive value 97.5%, specificity ~95%—although this radiographic sign appears in only ~25% of cases [27]. Lack of radiographic subluxation does not rule out partial tears, but its presence strongly indicates a complete rupture [27]. For more subtle instability, stress radiography under sedation can demonstrate tibial translation: tibial compression radiographs have long been shown to detect partial tears with high sensitivity (early work reported ~97% sensitivity) by revealing forward tibial thrust when axial load is applied [27]. Modern digital radiography allows precise measurement of cranial tibial translation on stress views, improving objectivity [27].

Rotational stress radiography has seen recent innovations. A 2023 study introduced a custom 3D-printed positioning device to apply standardized internal rotation torque during radiography [29]. Using this device, cadaveric stifles with transected CCL showed significantly greater lateral displacement of the calcaneus on torque-applied (stress) views compared to intact knees [29]. A calcaneal shift of ~3.45 mm under stress distinguished CCL-deficient limbs from normal with 87.5% sensitivity and 68.7% specificity [29]. In other words, measured lateral movement of the calcaneus on a stress radiograph strongly correlates with internal rotational laxity due to CCL rupture. This technique quantifies instability in both craniocaudal and rotational planes. In the same study, the method also differentiated inherent tibial torsion—a calcaneus positioned ≥3.25 mm lateral on neutral X-ray indicated anatomic tibial torsion with high accuracy [29]. These findings highlight how digital stress radiography (with assisted positioning devices or fluoroscopic analysis) can objectively measure joint laxity. Indeed, 3D fluoroscopic kinematic studies confirm that CCL insufficiency causes consistent cranial tibial subluxation and internal rotation during weight-bearing [15]. Dynamic imaging of dogs walking on a treadmill has shown the tibia remains subluxated ~9–10 mm cranially at mid-stance in CCL-deficient stifles [15]. Such analyses underscore that stress radiography and fluoroscopy, especially with 3D reconstructions, are valuable for visualizing the instability that CCL rupture induces throughout the gait cycle. Overall, radiographic techniques—from simple lateral views to advanced stress and dynamic imaging—continue to evolve, increasing the sensitivity of radiography for diagnosing CCLD and characterizing the instability patterns it produces.

### 2.5. Computed Tomography (CT) and CT Arthrography

CT provides detailed cross-sectional imaging of the stifle joint, offering enhanced visualization of soft tissues, joint alignment, and bone morphology. It can detect subtle abnormalities in limb conformation, such as femoral torsion and tibial plateau angles, which may predispose dogs to CCLD [4,6,30]. CT imaging has been increasingly utilized to assess tibial plateau morphology and detect osteoarthritic changes associated with CCLD. Advanced 3D reconstructions provide detailed visualization of bone remodeling and joint instability, assisting in preoperative planning for surgical correction. CT is also useful for evaluating coexisting conditions like meniscal damage and osteoarthritis, aiding in comprehensive treatment planning [4,6,30].

Modern multi-slice CT provides detailed osseous imaging of the stifle and is increasingly used in preoperative planning and research on CCL disease. One application is a precise measurement of bone geometry risk factors like the tibial plateau slope (TPS) and femoral alignment. CT can generate 3D reconstructions of the tibia, eliminating superimposition and improving measurement repeatability. A recent study comparing CT to conventional radiography for TPLO planning found that CT-based measurements of the tibial plateau angle (TPA) had superior intra- and inter-observer consistency and emerging deep learning models are being developed to automate TPA detection in radiographs with high accuracy [31,32]. In that study, observers measuring TPA on 3D CT images showed higher intraclass correlation coefficients than on radiographs, indicating more reliable and reproducible values [31]. Although mean preoperative planning angles were similar between CT and X-ray, CT’s ability to easily identify anatomic landmarks (tibial axes, plateau tangent) improved agreement between observers [31]. Thus, incorporating CT in surgical planning can reduce variability and ensure the intended post-osteotomy angle is achieved. CT is also invaluable for assessing tibial torsion or femoral anteversion—factors that may influence CCL strain—by allowing 3D angle measurements that are difficult on radiographs [27,28,33]. Normative data for femoral anteversion angles in dogs have been established via CT (e.g., ~31 in large breeds) [33], which can serve as a reference when evaluating dogs predisposed to cruciate rupture or patellar luxation.

Another major advance is CT arthrography (CTA), where iodinated contrast is injected into the joint before CT scanning. CTA improves the visualization of soft tissues by outlining structures in contrast fluid. In canine stifles, CTA markedly enhances the delineation of the cruciate ligaments and menisci compared to plain CT [28,34]. In an ex vivo study of normal dog stifles, CTA could even detect tiny linear contrast tracks within an otherwise intact CCL [28]. Notably, contrast leakage into the CCL substance was seen in 4 of 10 normal stifles, and histology confirmed micro-tears or clefts in 3 of those [28]. One ligament had a partial thickness tear (~5–10%) that was not grossly obvious but was revealed by contrast imbibition [28]. This demonstrates CTA’s high sensitivity for detecting partial ruptures: even a minor fiber disruption can allow contrast to percolate into the ligament, creating a visible signal of injury [34]. Clinically, CTA has been shown to identify partial CCL tears as a reduction in ligament cross-sectional area or contrast within the ligament [35]. It is also useful for evaluating meniscal tears—the contrast will outline displaced meniscal fragments or extrude into meniscal clefts [34]. In dogs with CCLD, CTA can delineate bucket-handle tears of the medial meniscus that might be missed on plain CT or radiographs [35].

Beyond diagnosis, CT is playing a growing role in 3D surgical planning and even custom implant fabrication. Patient-specific 3D bone models can be printed from CT scans, allowing surgeons to simulate osteotomies or design cutting guides. In the context of TPLO and other corrective osteotomies, researchers have developed CT-based 3D printed guides to improve surgical accuracy [36,37,38]. In a cadaveric trial, a customized 3D-printed drill/osteotomy guide for TPLO significantly reduced surgical time and variability in cut orientation [37]. TPLOs performed with the guide achieved postoperative TPAs closer to target 5 with less deviation in slope or rotation, compared to the freehand technique [37]. The guided group also had more consistent osteotomy inclination and fewer tibial tuberosity fractures, indicating the cut was positioned optimally [37]. These findings highlight the benefit of CT-guided planning: a virtual TPLO can be performed on the computer, the cutting plane and drill trajectory optimized, and then a guide is printed to replicate that plan on the patient. Such guides have been used for combined procedures (e.g., double-leveling osteotomies) as well [36,37]. Additionally, 3D-printed anatomical models (tibia with intact or torn menisci) have been used to study meniscal visibility and instrument access before and after TPLO [39]. As an example, one 2024 study laser-scanned a dog stifle and printed it with transparent menisci; it showed how the tibial plateau rotation of TPLO affects arthroscopic meniscal visualization [39]. The intersection of CT imaging with 3D printing and modeling is an exciting area that enhances both our understanding of CCLD biomechanics and the precision of surgical interventions [38]. In summary, CT and CT arthrography offer detailed insight into stifle anatomy and pathology—from subtle partial ligament tears to bone alignment—and are increasingly integral to sophisticated diagnostic and surgical workflows in CCL disease.

### 2.6. Magnetic Resonance Imaging (MRI)

MRI is unmatched in its ability to evaluate soft tissue structures, making it particularly useful for detecting partial tears and meniscal injuries [4,19,40]. Multiplanar imaging enhances diagnostic accuracy for subchondral bone changes, ligament integrity, and synovial thickening [4,40]. MRI allows for the detailed assessment of intra-articular structures, including early degenerative changes in the CCL before rupture occurs. Studies have demonstrated that MRI can identify ligament fiber disorganization and edema, which may serve as early indicators of ligament pathology. Despite its cost and accessibility limitations, MRI facilitates precise surgical planning and postoperative monitoring [40].

MRI provides unparalleled soft tissue contrast, making it a logical modality for directly visualizing the cranial cruciate ligament and associated structures. High-field MRI (1.5 T or higher) can depict the CCL as a distinct band within the intercondylar fossa; in cases of rupture, MRI can show fiber discontinuity, ligament retraction, or edema within the ligament substance [41,42]. Optimizing sequences is key for ligament imaging—proton-density (PD) and T2-weighted sequences (often with fat suppression) are commonly used. In developing an MRI protocol for canine stifles, researchers found that PD-weighted images provided the best depiction of normal cruciate ligaments and menisci [42]. These findings underscore the importance of sequence selection in achieving reliable and reproducible diagnostic results when assessing CCL integrity using MRI [29,41].

The CCL appears as low-signal (dark) on PD or T2 sequences, and tears are indicated by fluid signal (bright) within or in complete absence of the normal low-signal band. Short tau inversion recovery (STIR) sequences are particularly sensitive to bone marrow edema and joint effusion secondary to ligament injury. Interestingly, MRI studies have shown that dogs with even partial CCL tears can exhibit bone bruise patterns: one study reported high-signal STIR lesions in the femoral intercondylar notch or tibial eminence in 5 of 6 dogs with CCL disease (including partial tears and synovitis) [43,44]. These bone marrow lesions on STIR, analogous to those seen in human ACL injuries, suggest occult microfracture or stress from instability, reinforcing that MRI can detect secondary signs of CCL trauma (bone contusions) even in the early stages [43].

When it comes to diagnostic performance, MRI is quite accurate for confirming complete ruptures and can identify many partial tears, though distinguishing partial vs. complete tears is somewhat less reliable. A high-field (3 T) MRI study of dogs with known CCL ruptures showed MRI correctly identified all ruptured CCLs and even detected some subclinical fiber damage not noted during surgery [28]. In terms of sensitivity for partial tears, a presurgical 1.5 T MRI study reported about 85.7% sensitivity and 75% specificity in distinguishing partial versus complete CCL ruptures when compared to arthroscopy [42]. In other words, MRI missed some partial tears or misclassified them but overall had good agreement with surgical findings. MRI was also able to pick up meniscal tears with high accuracy in that study [42]. By contrast, low-field MRI (≤0.5 T) has more limitations. A classic study using a 0.5 T system for stifles found only ~64% sensitivity for meniscal tears versus the arthroscopic gold standard [35]. Low-field MRI tended to miss subtle meniscal damage, leading the authors to caution that a negative low-field MRI doesn’t exclude a tear [35]. They did note a decent specificity (~90%), meaning when low-field MRI showed a clear tear, it was usually correct [35]. Subsequent work improved low-field techniques (e.g., positioning the stifle at 90° flexion to better visualize the CCL on sagittal images) [43], but high-field MRI remains superior for fine details.

MRI can also evaluate meniscal pathology and cartilage in CCLD. Gradient-echo or T2* sequences can show meniscal tears as linear high-signal within the normally low-signal meniscus. One study found a sensitivity of ~92% for MRI-detecting meniscal tears when compared to arthroscopy [28], although this was under optimal conditions. Furthermore, MRI can grade cartilage thinning or defects and assess subchondral bone—features of osteoarthritis secondary to CCLD. Advanced MRI techniques, such as T2 mapping or dGEMRIC (delayed gadolinium enhancement for cartilage), have been explored in research dogs to quantify cartilage degeneration. Even gadolinium contrast MRI has been piloted to detect synovial enhancement or early osteophytes in CCLD, potentially highlighting active synovitis or early-stage OA changes that plain radiographs can’t see. In a recent exploratory study, an intra-articular gadolinium MRI was able to outline subtle meniscal tears and gave a contrast “highlight” to areas of ligament degeneration, though this is not routine clinical practice [43].

Lastly, MRI serves as a baseline for comparison with other modalities. For example, ultrasonography and CT arthrography techniques are often benchmarked against MRI or arthroscopy for validation. As MRI technology progresses, 3 T scanners and faster sequences are being applied to stifle imaging. A short-duration 3 T protocol in one study took only ~5 min per knee but still accurately diagnosed cruciate and meniscal tears in dogs [28]. This hints at a future where MRI could be a more rapid screening tool. And while cost and anesthesia requirements currently limit veterinary MRI use, its ability to non-invasively visualize all internal structures of the stifle—ligaments, menisci, cartilage, bone marrow, and synovium—makes it a gold standard for comprehensive evaluation of CCL injuries. High-field MRI is likely the most sensitive imaging method for partial ruptures short of arthroscopy, and ongoing improvements in technique and availability will continue to bolster its role in diagnosing CCL disease.

### 2.7. Ultrasonography and Elastography

Ultrasonography offers non-invasive, real-time imaging of soft tissue structures, aiding in the diagnosis of conditions like meniscal tears and joint effusion [4,40]. While its sensitivity and specificity can vary, ultrasonography is particularly effective for visualizing the cranial-distal portion of the CCL in larger dogs [4,40]. Ultrasonographic techniques offer a non-invasive method to evaluate ligament stiffness and fiber integrity. Elastosonography, in particular, provides quantitative data on ligament elasticity, aiding in the detection of early degenerative changes that are not visible on conventional radiographs. Although not a standalone diagnostic tool, it complements other modalities to provide a more comprehensive assessment of the stifle joint [4,40].

Musculoskeletal ultrasound of the stifle is gaining traction as a non-invasive tool to detect partial and complete CCL ruptures, especially in cases without obvious instability on an exam. Proper probe placement (usually cranial-parapatellar or lateral approaches with the knee flexed) and use of a high-frequency linear transducer (≈5–18 MHz) are critical to visualize the hyperechoic ligament fibers amid surrounding fat. A recent clinical study of dogs with stable (drawer-negative) stifles compared ultrasound to arthroscopic findings: ultrasonography correctly identified all cases of CCL pathology (100% sensitivity) but had some false positives (specificity ~58%) [45]. In that series, scanning was obtained with an 18–5 MHz linear probe and revealed fiber discontinuity or irregular ligament architecture in partial tears. The high negative predictive value (100%) suggests a normal ultrasound virtually rules out significant CCL damage [45]. These results indicate that MSK ultrasound is very sensitive for partial CCL injuries and can be a useful screening test in dogs without gross instability [45]. However, due to moderate specificity, positive ultrasound findings may need confirmation by arthroscopy [45].

To maximize accuracy, protocols recommend scanning both the insertion on the tibia (normally visible as parallel echogenic fibers for ~30–50% of the ligament length) and the mid-ligament area (often less clearly seen due to the overlying fat pad) [28]. Techniques like dynamic saline injection into the joint (ultrasound arthrography) can improve visualization: by distending the joint with fluid, the hyperechoic CCL is contrasted against anechoic saline, making partial tears (“fluttering” fiber ends and fluid clefts) more apparent [46]. This “contrast-enhanced” approach was shown in experimental studies to reveal ligament disruption that was otherwise obscured by the fat pad [46].

Beyond grayscale ultrasound, advanced ultrasonographic techniques are emerging for ligament assessment [47]. Shear-wave elastography (SWE) and strain elastography measure tissue stiffness, which can reflect ligament integrity [47]. Recent elastosonography studies in dogs have focused on the patellar ligament (an analog of the human patellar tendon) as an indirect marker of stifle mechanics post-CCL rupture [47]. One 2024 study using real-time strain elastography found that dogs with chronic CCL tears had a significantly thicker and stiffer patellar ligament compared to normal dogs, with progressive increases in ligament hardness the longer the CCL had been ruptured [48]. In early CCL disease, the patellar ligament elastogram is soft (red coloration), similar to healthy dogs, but over time it loses elasticity and becomes firmer (blue-green on elastography) [48]. These changes correlated with the chronicity of lameness, suggesting that secondary ligament/tendon overload occurs with long-standing instability. Such findings imply elastography could potentially serve as a noninvasive monitor of stifle pathology severity or duration. Similarly, acoustic radiation force impulse (ARFI) elastography has been tested on stifle joints (in research settings) to detect early meniscal or ligament changes. Preliminary reports indicate ARFI elastography is feasible and shows promise in identifying softening or tears in CCL and menisci before they are grossly apparent [43].

Ultrasound can also complement or be validated against MRI: in one study, high-frequency ultrasound delineated roughly the distal one-third of the intact CCL, and CTA/MRI was used to confirm the portions visualized [28]. Moreover, Doppler and contrast-enhanced ultrasound techniques (using microbubble contrast agents) are being explored to evaluate synovial perfusion and inflammation in CCL disease. While not yet routine, contrast-enhanced ultrasound (CEUS) may help identify synovial hyperemia or microvascular changes associated with partial tears and osteoarthritis.

In summary, ultrasonography—with optimized settings and possibly adjuncts like elastography or injected contrast—has shown high sensitivity for CCL ruptures and is entirely noninvasive. It can detect partial tears that elude a physical exam, and emerging modalities enable functional assessment of ligament stiffness. This makes ultrasound an attractive diagnostic technique and adjunct to orthopedic exams, especially for early or partial CCLD cases where radiographs are equivocal and MRI is not readily available.

## 3. Surgical Treatment

### 3.1. Extra-Articular Stabilization

Lateral Fabellar Suture Stabilization (LFSS) A popular treatment for CCLD is the lateral fabellar suture stabilization procedure (LFSS), which involves suturing the tibia from the lateral fabella to counteract cranial tibial thrust [49,50,51]. In a study comparing bone anchor stabilization (BAS) with LFSS, it was discovered that BAS offered a more robust attachment with noticeably less displacement, but LFSS displayed greater displacement under physiological loading, indicating possible instability [50]. Specifically, BAS yielded a median displacement of 0.91 mm, indicating a more reliable fixation point, while LFSS showed a median total movement of 2.17 mm [50]. This study emphasizes how crucial it is to put sutures correctly around the femoro-fabella ligament because poor execution can lead to inconsistent LFSS results [50]. As a result, BAS might be a more reliable choice for surgeons looking to improve joint stability [50].

### 3.2. Intra-Articular Stabilization

There are two main types of intra-articular stabilization procedures: natural (over-the-top) treatments and synthetic implants. Natural methods use the dog’s own tissues for stability, usually by mimicking the function of the CCL with a strip of the patellar ligament or other nearby structures, such as fascia lata [7]. This biologically integrated approach may lower the risk of problems related to foreign materials while also supporting joint stability and healing [7,22]. On the other hand, synthetic implants imitate the mechanical properties of the CCL by using synthetic materials like polyester or polypropylene [31,52]. The purpose of these implants is to give the stifle joint instant mechanical support, which is crucial when there has been a large ligament rupture. The goal of both synthetic and natural approaches is to improve joint stability and clinical results for canines with CCLD [31,52,53]. While synthetic implants can provide instant stabilization, natural approaches encourage a more natural connection [7].

The intra-articular reconstruction of the CCL by an organic graft or a synthetic implant allows for the restoration of physiological stifle stability [5]. A synthetic implant, such as ultra-high-molecular-weight polyethylene (UHMWPE), can be positioned under arthroscopic guidance and fixed with interference screws through femoral and tibial bone tunnels [5]. This technique has shown promising outcomes, including rapid weight-bearing, minimal muscle atrophy, and stable craniocaudal translation postoperatively [5]. Radiographs have indicated congruent articular surfaces without worsening of osteoarthrosis over the follow-up period [5]. This method may be considered an alternative for treating CCL rupture in large dogs, pending further prospective studies [5].

In the end, the decision between these approaches may be influenced by factors including the dog’s particular demands, the severity of the damage, and the surgeon’s experience [4,8,22]. Sufficient investigation into these methods is necessary to maximize therapeutic approaches and guarantee long-term success.

### 3.3. Tibial Plateau Leveling Osteotomy (TPLO)

Tibial plateau leveling osteotomy (TPLO) is a surgical treatment utilized to stabilize the CCL-deficient stifle [23,26,54]. It primarily addresses the biomechanical alterations brought on by ligament damage. The tibial plateau is reshaped or modified during the procedure to change its geometry; normally, this results in a 5–7-degree reduction in the natural slope of the tibial plateau, which can vary from 25 to 30 degrees in dogs [24,26]. Therefore, this modification is even more indicative in CCL-deficient stifles with associated steep tibial plateau angles. Such a modification seeks to reduce the requirement for the CCL to prevent cranial tibial thrust by changing the joint mechanics from a sliding hinge to a more stable sliding platform [11,26]. The tibial plateau is cut and rotated to provide a more neutral angle, usually fixed at about 90 degrees with respect to the long axis of the corresponding tibia [26,51]. This modification helps to stabilize the joint by preventing the tibia’s aberrant forward movement while bearing weight (i.e., cranial tibial thrust) [5,26]. A bone plate and screws are used to anchor the repositioned tibial plateau, enabling rapid weight-bearing following surgery and accelerating the healing process [7]. To enhance optimal healing and maximize recovery, physical therapy and restricted exercise are implemented [8].

A recent advancement in TPLO is a modified tibial plateau leveling osteotomy technique known as double-cut tibial plateau leveling osteotomy (DCTPLO) [10]. This technique entails making two cuts in the same plane to more precisely level the tibial plateau [10]. A study involving 18 stifles in dogs with excessive tibial plateau angle (eTPA > 34°) demonstrated that DCTPLO effectively reduced eTPA levels, with preoperative and postoperative tibial plateau angle values decreasing from a mean of 39.4° to 6.3° [10]. The technique was found to be feasible and reproducible, with a majority of stifles achieving radiographic healing within 60 days and only minor complications reported [10]. The TPLO procedure is frequently preferred for its ability to provide medium-to large-breed dogs with long-term stability and functional improvement; however, the technique requires specific training and equipment for perfect execution.

### 3.4. Tibial Tuberosity Advancement (TTA)

Tibial tuberosity advancement (TTA) is a surgical procedure for managing cranial cruciate ligament (CCL) injuries, aiming to reduce cranial tibial thrust by altering the biomechanics of the stifle joint [5,8]. Unlike tibial plateau leveling osteotomy (TPLO), which modifies the tibial plateau angle, TTA advances the tibial tuberosity, changing the patellar ligament’s angle of pull to approximately 90°. This adjustment enhances the quadriceps mechanism’s ability to resist cranial tibial thrust in a CCL-deficient stifle [8,19]. By advancing the tibial tuberosity, the load-bearing pressures on the joint are redistributed, reducing reliance on the CCL for stabilization during movement [8,19]. Additionally, neutralization of tibiofemoral shear forces occurs at a patellar ligament angle of 90.3 ± 9.0, further contributing to joint stability [26].

In the TTA procedure, the tibial tuberosity is surgically cut, advanced, and stabilized using an implant or cage [19,55]. This approach addresses cranial tibial subluxation, promoting joint stability and recovery. To optimize postoperative outcomes, rehabilitation protocols emphasize progressive physical therapy and controlled exercise to restore joint function [3,8,49]. As a less invasive alternative to TPLO, TTA is often considered suitable for dogs of various sizes while effectively addressing the mechanical instability associated with CCL injuries [8,19,55].

The choice between TPLO and TTA depends on factors such as the dog’s size, activity level, and anatomical characteristics. A comprehensive evaluation of the dog’s clinical presentation and history is essential to determine the most appropriate surgical method for achieving optimal outcomes and long-term joint health [3,19].

### 3.5. Stifle Arthroscopy

Stifle arthroscopy has emerged as a minimally invasive and precise technique for diagnosing and managing CCL disease (CCLD) [4,19]. This approach provides detailed visualization of the joint, facilitating accurate diagnosis and targeted intervention. Arthroscopy is often combined with stabilization techniques such as lateral fabellar suture stabilization, TPLO, or TTA to enhance treatment efficacy [4,19].

By allowing direct assessment of intra-articular structures, including the CCL, menisci, and articular cartilage, arthroscopy improves surgical outcomes and postoperative recovery. Studies suggest that combining TPLO with arthroscopy and lateral fabellar suture stabilization may yield superior joint stability and functional recovery, leveraging the strengths of each technique [4,19].

Diagnostic arthroscopy of the stifle is considered the reference standard for confirming CCL tears and associated intra-articular injuries. With miniaturized scopes (2.7–4 mm) and improved instrumentation, veterinary arthroscopy can directly visualize the cranial cruciate ligament—often revealing fraying, partial tearing, or complete discontinuity—and probe both menisci for tears [5,8]. Numerous studies have demonstrated arthroscopy’s superior sensitivity for meniscal tears. For example, in one comparison, arthroscopy detected significantly more medial meniscus tears than open arthrotomy did, especially small caudal horn lesions [41,55]. Compared to low-field MRI, arthroscopy allows for higher-resolution visualization of subtle intra-articular pathology and can facilitate immediate intervention during CCL stabilization surgery [42,50].

Relative to imaging, arthroscopy outperforms MRI and CT in identifying fine meniscal damage: low-field MRI misses ~36% of meniscal tears as noted, whereas an experienced arthroscopist can directly see and feel tears that subtle imaging signs might overlook [35]. Thus, for dogs with CCL rupture, arthroscopic inspection of the joint remains the gold standard to definitively diagnose meniscal injury (and to a lesser extent to confirm partial vs. complete CCL tears) [35]. This high accuracy is why many surgeons now perform routine arthroscopy at the time of CCL surgery—it allows immediate treatment of any meniscal tears found. In fact, “arthroscopic meniscectomy” or meniscal release, can be performed in the same session, avoiding the need for a second surgery. Recent evidence suggests arthroscopy can identify meniscal pathology earlier and more comprehensively than even high-field MRI in some cases, reinforcing its diagnostic value [55].

Beyond diagnosis, arthroscopy has evolved into a therapeutic tool for CCL injuries. New techniques enable arthroscopic-assisted ligament reconstruction in dogs, analogous to human ACL surgery. For example, one case report describes the arthroscopic placement of a synthetic ligament (ultra-high-molecular-weight polyethylene) to replace a torn CCL [5]. Through a few small incisions, the surgeons performed full diagnostic arthroscopy (confirming a complete CCL rupture and a minor meniscal tear), debrided the remnants with an arthroscopic shaver, and then drilled bone tunnels in the femur and tibia under arthroscopic guidance to pass the synthetic graft [5]. The graft was secured with interference screws, with minimal invasiveness. At 6-month follow-up, the dog had returned to normal function with no progression of OA [5]. This demonstrates that minimally invasive CCL reconstruction is feasible—arthroscopy ensures accurate tunnel placement at the ligament footprints and minimizes trauma to surrounding tissues.

Another development is arthroscopy-guided “tightrope” procedures or suture placements for extracapsular stabilization, where arthroscopy helps verify isometric points. Additionally, techniques to improve arthroscopic visualization, such as joint distraction via temporary clamps or saline pumps, have been studied [56]. One study showed that applying gentle traction to separate the joint surfaces significantly improved the arthroscopic view of the caudal meniscus, allowing more complete evaluation and repair [56]. These enhancements make arthroscopy an even more powerful approach for both diagnosing and treating intra-articular damage in CCLD.

It is worth noting that arthroscopy is not without a learning curve or equipment needs, but as it becomes more common, evidence consistently shows better outcomes for meniscal inspection and treatment. Dogs whose meniscal tears are addressed arthroscopically at the time of CCL stabilization tend to have reduced lameness and fewer subsequent “meniscal click” episodes. Some orthopedic centers report that arthroscopy has essentially eliminated the incidence of missing a meniscal tear at the initial surgery, which historically was an issue leading to persistent lameness. In comparison to open arthrotomy, arthroscopy causes less periarticular trauma and promotes quicker recovery of range of motion. One study even found arthroscopy had a higher detection rate of subtle meniscal tears than arthrotomy, highlighting that looking through a scope with magnification can reveal fine lesions that might be missed via a limited arthrotomy view [55].

In summary, arthroscopy remains the diagnostic gold standard for intra-articular lesions in CCLD. It surpasses imaging for fine-detail detection (particularly of meniscal pathology) and now serves as a minimally invasive platform for concurrent intervention—from meniscal trimming to experimental intra-articular ligament replacement. Ongoing advancements in arthroscopic instruments and techniques (e.g., angled scopes, better fluid management, joint distractors) continue to improve its utility. As a result, many surgeons advocate that arthroscopy be integrated into CCL treatment whenever possible to ensure no internal derangements go untreated. The latest literature cements arthroscopy’s role as both a diagnostic endpoint (against which other modalities are measured) and a therapeutic modality that can enhance recovery in canine CCL disease.

The discussed surgical procedures and associated outcomes are summarized in Table 3.

### 3.6. Emerging Biomarkers and Molecular Diagnostics

Beyond imaging, there is intense interest in biomarkers—biochemical indicators in blood or synovial fluid—that could signal early CCL degeneration or predict disease. CCL disease often has an inflammatory component, so cytokines and proteinases have been evaluated as potential biomarkers [13]. Recent studies have measured various mediators in the synovial fluid of dogs with CCL rupture. For instance, one 2020 study assessed interleukin-8 (IL-8), monocyte chemoattractant protein-1 (MCP-1), and keratinocyte-derived chemokine (KC, analogous to IL-8) in the joint fluid of dogs with CCL rupture versus healthy dogs [57]. All three inflammatory cytokines were significantly elevated in CCL rupture knees, correlating with the degree of synovitis on histopathology [57]. Notably, synovial fluid MCP-1 showed excellent diagnostic performance, with an AUC of 0.91 for distinguishing dogs with CCL-related osteoarthritis from controls [57]. Using a cutoff of ~265 pg/mL, MCP-1 could identify CCL/OA with ~85% sensitivity and 98% specificity in that study [57]. This suggests that MCP-1 is a promising biomarker of stifle inflammation due to CCL rupture. Elevated IL-8 and KC in synovial fluid also correlated with inflammatory cell infiltration scores [57], indicating they reflect active synovitis. In serum, however, traditional matrix metalloproteinases (MMP-2, MMP-3) did not significantly differ between CCL rupture dogs and controls in the same study [57], highlighting that local (joint) markers may be more sensitive than systemic ones. Other synovial markers under investigation include lubricin, which was found to increase in CCL rupture joints, possibly as a compensatory response [58], and acrolein (a byproduct of oxidative stress), which has been detected at higher levels in osteoarthritic joint fluid. The emerging picture is that a panel of synovial inflammatory mediators could serve as an early warning of CCL disease progression, even before severe instability occurs.

Genomic and proteomic approaches are also being applied. miRNAs (microRNAs), which are small non-coding RNAs that regulate gene expression, have garnered attention as both pathogenic factors and biomarkers in CCL disease. A 2022 pilot study performed comprehensive miRNA sequencing of stifle synovium from dogs with CCL rupture-induced OA versus healthy dogs [59]. They discovered over 50 miRNAs significantly upregulated and dozens downregulated in the diseased joints. Importantly, two microRNAs—miR-542 and miR-543—were consistently elevated not only in the synovial tissue but also in the synovial fluid and even circulating serum of CCL/OA dogs [59]. This consistency across tissue, fluid, and blood suggests these miRNAs could be minimally invasive biomarkers (detectable via blood test) for CCL degeneration and osteoarthritis. miR-542/543 are thought to be involved in inflammatory and catabolic pathways in the joint. This study [59] was the first to profile canine synovial miRNAs so extensively, and it opens the door to using liquid biopsy techniques (like measuring circulating miRNAs) to detect early joint disease.

Likewise, metabolomic profiling has been explored: researchers using NMR spectroscopy on synovial fluid found distinct metabolite changes in dogs with meniscal injury (e.g., increased mobile lipids) compared to those without, suggesting metabolite biomarkers for concurrent meniscal tears [17]. Another high-tech approach used Fourier-transform infrared (FTIR) spectroscopy combined with machine learning to analyze synovial fluid. The FTIR spectral “fingerprint” was able to accurately distinguish dogs with cruciate rupture OA from normal dogs, effectively serving as a biochemical screening tool [59]. Such spectroscopic methods could one day allow rapid point-of-care tests on a drop of joint fluid to diagnose CCLD and OA.

On the horizon, machine-learning algorithms are being developed to integrate these diverse data sources [60]. For example, a genomic prediction study applied Bayesian machine learning models to the genetic data of Labrador Retrievers to predict the risk of CCL rupture’. Identifying at-risk dogs genetically could inform preventative strategies. In diagnostics, AI is being explored to read medical images—while canine-specific AI for CCL is in the early stages, analogous human studies show promise. A deep learning CNN model was recently able to automatically detect ACL tears on human knee MRI with >90% accuracy [53]. It is conceivable that similar neural networks could be trained on veterinary MRI or even radiographs to flag cruciate tears or measure TPA automatically [53,60]. Additionally, combining imaging with biomarkers in a machine-learning model could improve overall diagnostic confidence. For instance, an algorithm that takes into account radiographic findings, a panel of synovial fluid cytokines, and perhaps gait analysis data might classify stifles as “partial tear”, “complete tear”, or “intact” with high accuracy—essentially an AI-driven decision support tool for CCLD. Some preliminary work in this direction includes using insurance big data and ML to predict orthopedic injuries [61] or unsupervised learning to stratify ACL injuries by severity [59].

In summary, the frontier of CCL diagnosis is moving toward multimodal biomarkers and AI integration. Cytokines like MCP-1 and IL-8 in joint fluid show potential for early, non-radiographic detection of ligament pathology. Novel molecules (miRNAs, metabolites) identified through omics technologies could become minimally invasive indicators sampled via blood or synovial fluid. These biomarkers, combined with advanced analytic techniques, might allow vets to diagnose CCL disease or meniscal injury before significant structural failure occurs. Machine learning and AI will likely play a role in synthesizing imaging findings with biomarker profiles to improve the classification and prognostication of CCL injuries. While these approaches are still largely in the research phase, they underscore a shift toward a more comprehensive, precision-medicine approach in veterinary orthopedics—where a blood test or fluid analysis, aided by AI, could complement imaging to provide a robust, early diagnosis of cranial cruciate ligament disease in dogs [17,53,57,58,59,62].

### 3.7. Postoperative Rehabilitation

Postoperative rehabilitation is a critical component of CCLD management, focusing on progressive exercise regimens to promote early mobilization, regardless of the surgical method employed [3,8,19,49]. Rehabilitation aims to restore muscle strength, joint range of motion, and proprioception through controlled weight-bearing exercises, thereby improving joint stability and reducing postoperative complications [3,8].

Physical therapy modalities such as hydrotherapy, therapeutic exercises, and passive range of motion (PROM) are tailored to the individual needs of each patient, ensuring a safe and effective return to normal activities [8,20,49]. Current rehabilitation strategies for postoperative care are summarized in Table 4.

A study comparing functional and radiographic outcomes in dogs undergoing TPLO or lateral fabellar suture stabilization (LFS) revealed significant improvements in limb peak vertical force following identical postoperative rehabilitation regimens [49]. Both methods demonstrated substantial functional recovery, highlighting the importance of comprehensive rehabilitation in achieving optimal outcomes [8,20,49]. Despite an increase in osteoarthrosis (OA) scores from preoperative to 24 months, no significant differences were observed between the TPLO and LFS groups, reinforcing the efficacy of these procedures when combined with rehabilitation [2,49].

Specific rehabilitation interventions have also been evaluated. Marsolais et al. conducted a prospective controlled trial comparing dogs undergoing postoperative rehabilitation with those subjected to activity restriction after extracapsular repair [33]. Rehabilitation included swimming, PROM, therapeutic walking, and massage, resulting in significantly improved peak vertical force and vertical impulse at six months post-surgery compared to the activity-restriction group [20]. While swimming enhanced muscle strength and cardiovascular fitness, access to facilities limited its feasibility for some patients. This study, although promising, was classified as level III evidence due to methodological limitations such as lack of blinding [20].

Cold compression therapy (CCT) has also shown benefits in reducing postoperative pain and swelling. Studies by Rexing et al. and Drygas et al. demonstrated that CCT effectively improved pain scores and reduced limb circumference within 24 h post-surgery, supporting its inclusion in postoperative care protocols [20].

Long-term monitoring is essential to maintain joint health, assess surgical success, and adapt rehabilitation strategies to address potential complications, such as implant failure or OA progression [2,20,49,54]. By combining advanced surgical techniques with tailored rehabilitation programs, optimal recovery and quality of life can be achieved for dogs with CCL injuries.

## 4. Conclusions

Dogs who suffer from cranial cruciate ligament disease (CCLD) experience severe impairments to their mobility and general well-being. The complexity of this illness is due to a combination of hereditary, mechanical, and physiological variables [1,3,8]. Effective diagnosis and treatment of stifle joint dysfunction require a detailed understanding of the structure of the joint, the function of the cranial cruciate ligament (CCL), and joint dynamics [8,19,63]. The nature of the disease is determined by its pathophysiology and etiology, which are influenced by contributing factors such as age, breed, obesity, and abnormal body shape [1,3,8,24]. By appropriate action, early detection of these factors can help prevent long-term damage and improve the quality of life for affected dogs [1,3,8,24].

Developing suitable treatment strategies requires evaluating the condition using a variety of procedures, such as physical exams, X-rays, and advanced imaging modalities like MRI [4,8,19]. Combining surgical techniques including tibial plateau leveling osteotomy (TPLO), tibial tuberosity advancement (TTA), and arthroscopy with rehabilitation regimens has shown encouraging outcomes [4,8,19]. With the goal of improving general function, minimizing discomfort, and regaining joint stability, these procedures represent advancements in orthopedic therapy. Further research into the root causes, potential risks, and advanced treatment methods is essential [1,3,8]. Gaining insights can improve treatment outcomes and aid in developing preventive measures to reduce the occurrence and impact of CCLD in dogs [1,3,8]. By combining various approaches and advancements in care, we can more effectively enhance the well-being and mobility of dogs affected by CCLR. Nevertheless, the progression of stifle osteoarthritis following an individual or combined surgical procedure(s) remains questionable and requires further long-term investigation and monitoring to address such a crucial issue [3,23,54].

## Figures and Tables

**Table 1:** Breed Differences in Canine CCLD: This table compares large and small breed dogs regarding their risk of developing CCLD, commonly affected breeds, and management considerations.

Characteristic	Large Breed Dogs	Small Breed Dogs
Risk of CCLD	Higher due to greater body weight and joint stress	Lower, but can still occur due to conformation issues
Common Breeds Affected	Labrador Retriever, Rottweiler, Saint Bernard, Newfoundland	Shih Tzu, Yorkshire Terrier, Miniature Poodle
Joint Conformation	Increased tibial plateau angle, leading to higher shear forces on the CCL	More prone to patellar luxation, affecting ligament stability
Primary Contributing Factor	Degenerative changes due to chronic stress and weight-bearing forces	Tibial instability secondary to patellar luxation (especially Grade IV cases)
Management Approach	More likely to require surgical intervention (TPLO, TTA)	Conservative management may be more effective, but surgery is indicated in severe cases
Postoperative Recovery	Longer due to higher mechanical loading on joints	Typically faster, but risk of complications from concurrent orthopedic issues (e.g., patellar luxation)

**Table 2:** Predisposing Factors for Canine CCLD: This table summarizes key factors contributing to cranial cruciate ligament disease (CCLD) in dogs, including breed predisposition, conformation, obesity, genetics, and immune-mediated mechanisms.

Key Factor	Description
Breed	Large breeds (e.g., Labrador Retrievers, Rottweilers, Saint Bernards) have a higher risk due to joint stress. Small breeds may develop CCLD due to tibial instability (e.g., patellar luxation).
Conformation	Breeds with hyperextended pelvic limbs and open joint angles (e.g., Rottweilers, Chow Chows) have increased ligament strain. Hip dysplasia worsens stress.
Obesity	Excess weight increases joint stress and inflammation, accelerating ligament degeneration. Genetic variations in ADIPOQ may affect ligament integrity.
Genetics and Parity	Parous female Rottweilers have a significantly lower risk, suggesting a protective hormonal or mechanical factor.
Immune-Mediated	Autoimmune responses and inflammatory mediators (e.g., MMPs) contribute to ligament breakdown.
Inactivity	Muscle atrophy and poor neuromuscular control reduce joint stability, increasing ligament strain. Proprioceptive training helps mitigate risk.

**Table 3:** Summary of Surgical Techniques for Treating Canine CCLD: This table outlines key surgical methods used to treat CCLD in dogs, highlighting each technique’s description and associated outcomes.

Technique	Description	Outcomes
Extra-Articular Stabilization	Lateral fabellar suture technique involves the placement of a suture from the femur to the fibula to stabilize the stifle.	Effective for stabilization but may not address underlying bone deformities or long-term outcomes.
Intra-Articular Stabilization	Utilizes natural or synthetic implants to reconstruct the cranial cruciate ligament.	Good short-term results; choice between natural vs. synthetic implants depending on specific cases.
TPLO	Reshapes the tibial plateau to alter the biomechanics of the stifle joint, reducing tibial thrust.	Effective in reducing tibial thrust and improving function; requires accurate preoperative planning.
DCTPLO	Modified TPLO technique involving two cuts in the same plane to level the tibial plateau.	Effective for cases with excessive tibial plateau angle; significantly reduces eTPA with good clinical outcomes.
TTA	Shifts the tibial tuberosity to reduce patellar tendon force on the cranial cruciate ligament.	Improves function and reduces lameness; may have a longer recovery period compared to TPLO.
Arthroscopy	Minimally invasive technique to visualize and treat the joint, often used in combination with other surgical methods.	Allows for detailed assessment and treatment of intra-articular issues; can be combined with TPLO or other techniques.

**Table 4:** Summary of Surgical Techniques: Rehabilitation strategies for postoperative canine cranial cruciate ligament disease: efficacy, advantages, and limitations.

Rehabilitation Method	Description	Advantages	Disadvantages
Aquatic Therapy (Swimming)	Engages dogs in swimming within a controlled aquatic environment, allowing for low-impact exercise that facilitates movement without stressing the joints.	– Minimally invasive approach to enhance cardiovascular fitness and muscle hypertrophy. – Facilitates greater range of motion due to buoyancy, reducing joint stress. – Can be tailored to individual tolerance levels and recovery phases.	– Requires access to specialized facilities or equipment, which may not be universally available. – Potential for fatigue or anxiety if not carefully monitored, particularly in less experienced swimmers. – Risk of water-related complications if hygiene and safety protocols are not observed.
Passive Range of Motion Exercises	Involves manual manipulation of affected joints to maintain or improve flexibility and prevent stiffness.	– Effective in preserving joint mobility and preventing fibrosis in the periarticular tissues. – Can be performed by owners with proper instruction, facilitating at-home care. – Requires no specialized equipment, promoting ease of accessibility.	– Efficacy may be limited without concurrent active exercise interventions. – Requires careful training of owners to ensure proper techniques are employed to avoid injury.
Underwater Treadmill Rehabilitation	Utilizes a treadmill submerged in water to facilitate controlled ambulation and resistance training.	– Provides an environment that minimizes joint impact while promoting weight-bearing exercise. – Adjustable parameters (e.g., water level, speed) allow for progressive loading and tailored rehabilitation protocols. – Effective in enhancing muscle strength and improving gait mechanics.	– High initial investment and maintenance costs associated with specialized equipment. – Limited availability of facilities equipped with underwater treadmills. – Some dogs may exhibit anxiety or reluctance in unfamiliar environments.
Therapeutic Exercise Programs	Structured exercise regimens are designed to target specific muscle groups and enhance functional recovery.	– Allows for individualized rehabilitation plans based on specific surgical interventions and patient needs. – Promotes muscle re-education and proprioceptive feedback mechanisms. – Can be adjusted in intensity and volume as recovery progresses, ensuring optimal outcomes.	– Requires oversight from trained rehabilitation professionals to avoid overexertion and ensure safety. – Limited efficacy if adherence to prescribed exercise regimens is lacking.
Cold Compression Therapy	Involves the application of cold packs combined with compression to reduce inflammation and manage postoperative pain.	– Provides immediate post-surgical pain relief and minimizes edema through vasoconstriction. – Non-invasive and easily administered, facilitating rapid implementation. – Can be employed in conjunction with other therapeutic modalities.	– Primarily offers symptomatic relief, with effects being transient and requiring repeated applications. – May necessitate owner education to ensure correct and safe application techniques.
Massage Therapy	Involves the manual manipulation of soft tissues to enhance circulation and alleviate muscle tension.	– Can improve lymphatic drainage and reduce muscle soreness, contributing to overall recovery. – May enhance the bond between the pet and owner, promoting emotional well-being. – Can be tailored to individual needs and preferences.	– Requires practitioners with specialized training to ensure effectiveness and safety. – The variability in individual responses limits the generalizability of outcomes.
Electrical Stimulation Therapy	Utilizes electrical currents to stimulate muscle contraction and promote rehabilitation.	– Facilitates muscle strengthening and pain management without excessive stress on healing tissues. – Effective in counteracting disuse atrophy in non-weight-bearing limbs. – Non-invasive, allowing for adjunctive use alongside other rehabilitation modalities.	– Equipment costs and the need for trained personnel may limit widespread application. – May not be suitable for all patients, especially those with contraindications for electrical stimulation.
Activity Restriction Protocols	Involves limiting physical activity to facilitate initial healing and mitigate the risk of postoperative complications.	– Reduces the likelihood of joint destabilization or injury during early recovery phases. – Allows for focused healing of surgical sites, promoting optimal surgical outcomes.	– Extended restriction may lead to muscle atrophy and joint stiffness if not managed with progressive rehabilitation strategies. – Potential psychological impacts on the dog due to limited mobility and activity.

## Data Availability

The original contributions presented in the study are included in the article, further inquiries can be directed to the corresponding author.
